# Bronchopleural fistula after non small cell lung cancer radiofrequency ablation: what it implying to us?

**DOI:** 10.1186/1746-1596-8-202

**Published:** 2013-12-10

**Authors:** Weimiao Li, Lijun Huang, Yong Han, Yongan Zhou, Qiang Lu, Xiaofei Li

**Affiliations:** 1 Department of Thoracic Surgery, Tangdu Hospital, Fourth Military Medical University, Xi’an 710038, China

**Keywords:** Non–small cell lung cancer, Radiofrequency ablation, Pathological changes

## Abstract

**Abstract:**

Radiofrequency ablation (RFA) is an alternative method to treat the inoperable NSCLC and there were few serious complications after RFA therapy have been reported. Here, we reported a NSCLC patient endured empyema after treatment by RFA for one month. There was a 20 × 25 × 20 mm mass on the right middle lobe by CT scan before RFA and a huge gas cavity with liquid was found in the right chest cavity after RFA treatment for twenty- eight days. A hole in the right middle lobe was found with large amount of pus in the pleural cavity as well as the bronchopleural fistula (BPF) during the operation. Results from the postoperative pathology showed a multiple small foci differentiated adenocarcinoma, partial bronchiolar-alveolar carcinoma, 0.5 cm away around the hole at the same time. It is difficult to diagnose and treat the rare complication of BPF, while, the larger field of ablation might be helpful to postpone the tumor local progression. Therefore, surgery was a good option for BPF especially when an empyema occurred.

**Virtual slides:**

The virtual slide(s) for this article can be found here: http://www.diagnosticpathology.diagnomx.eu/vs/8028049341122276.

## Background

Despite surgical resection was the standard treatment of localized non–small cell lung cancer (NSCLC), only 20% of all diagnosed lung cancers were suitable for potentially curative resection [1]. Radiofrequency ablation (RFA) was proved safe and effective for the treatment of inoperable NSCLC as an alternative method [2]. Few serious complications from RFA have been reported [3]. We reported a NSCLC patient who was first treatment by RFA and endured empyema one month after treatment.

## Case presentation

**Figure 1:**
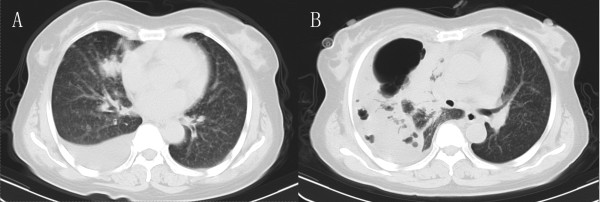
**Chest CT scans of preoperative and postoperative. A**: A chest CT scan showed a 20 × 25 × 20 mm mass on the right middle lobe as well as an amount of pleural effusion on admission. **B**: An emergent chest CT scan showed a huge gas cavity and liquid located in the up right chest cavity.

A 60 year-old woman was admitted to our hospital with complaints of chest pain for over 1 month, with chest tightness, shortness of breath for 7 days, without fever, cough, diarrhea and abdominal pain. On admission, a 20 × 25 × 20 mm mass on the right middle lobe as well as an amount of pleural effusion were showed by a chest computer tomography (CT) scan (Figure 1A). However, Bronchoscope was normal. The puncture biopsies confirmed adenocarcinoma cells about the lung mass. Tumor cells were not founded in the pleural effusion with the puncture examination.

**Figure 2:**
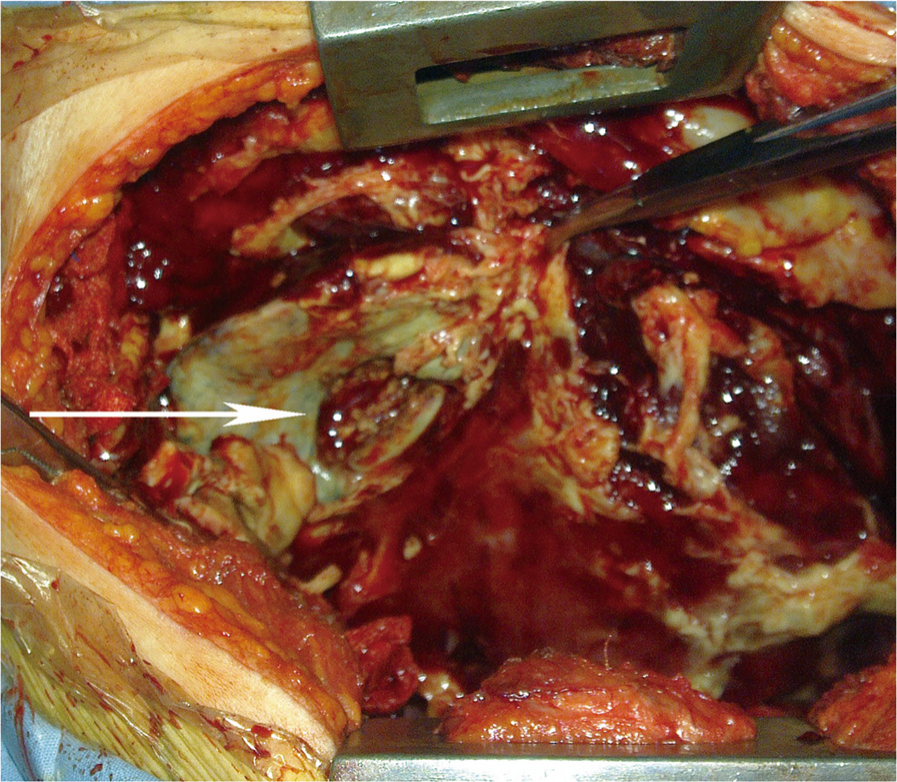
A hole in the right middle lobe was found with large amount of pus in the pleural cavity during the operation.

**Figure 3:**
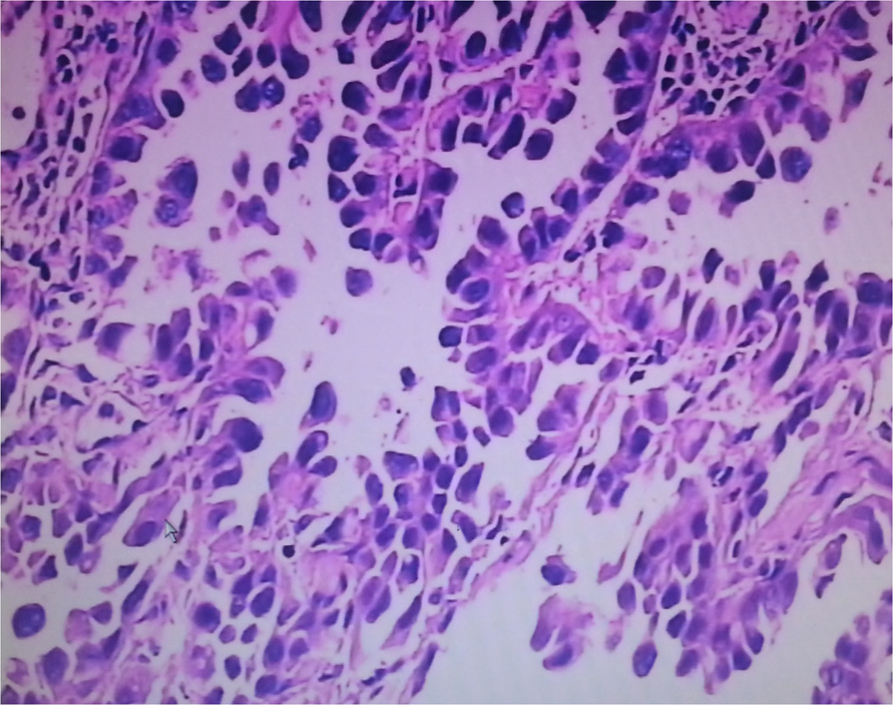
The postoperative pathology confirmed partial bronchiolar - alveolar carcinoma at the edge of hole(× 200).

**Figure 4:**
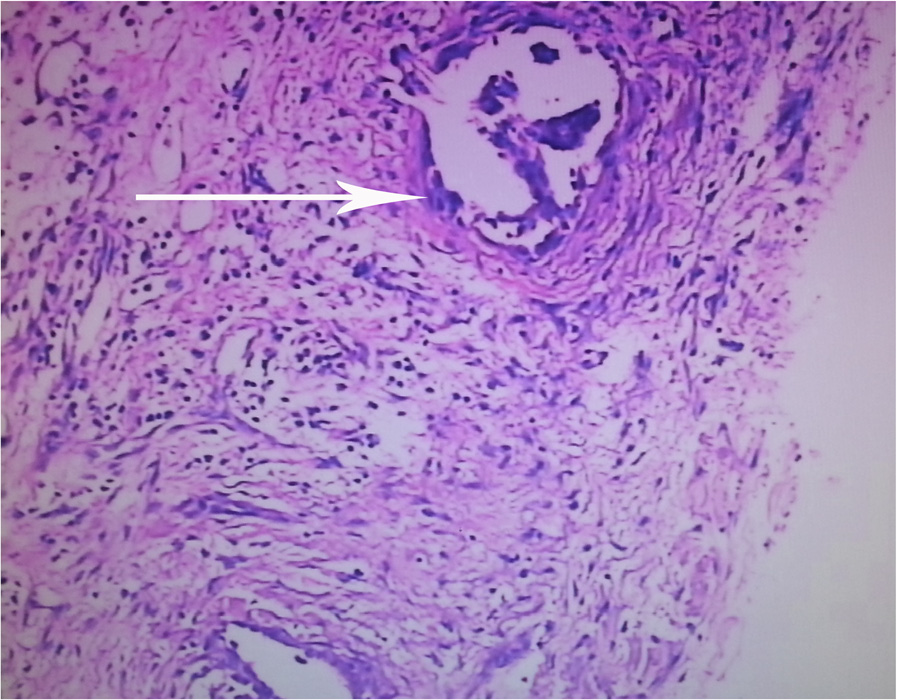
Multiple small foci differentiated adenocarcinoma was founded at the edge of hole after operation(×100).

Patient refused surgery and radiofrequency ablation was performed then. The patient recovered rapidly and discharged 5 days after RFA treatment. The patient was readmitted because of chest tightness, shortness of breath and fever at 38.5°C twenty-three days after discharge. The number of her leukocytes in blood increased to 22 × 109/L. An emergent chest computer tomography (CT) scan showed a huge gas cavity and liquid located in the up right chest cavity (Figure 1B). Empyema was confirmed by thoracentesis and thoracotomy was carried out thereafter. During the operation, we found a hole in the right middle lobe with large amount of pus in the pleural cavity (Figure 2). The resected right middle lobe showed a 30 × 35 × 32 mm hole perforating into lobe and communicating with bronchus. The bronchopleural fistula (BPF) was found and the whole layer of visceral pleura shown thickened (Figure 2). The postoperative pathology confirmed the multiple small foci differentiated adenocarcinoma and partial bronchiolar - alveolar carcinoma at the edge of hole (Figures 3,4). The postoperative evolution was uneventful and patient was discharged in 7th days after operation.

## Discussion

In recent years, the treatment of NSCLC is still not satisfying in spite of the rapid development of lung cancer research [4,5]. In addition to the use of chemotherapy drugs [6], RFA is a potential local therapy used for NSCLC patients who were not suitable for surgery or refused operation [7,8]. Several authors have assessed the performance of RFA in treatment for primary NSCLC and the complications of RFA were believed to be acceptable [9-11]. Our previous studies found that complications of RFA included pneumothorax (19.1%), hemoptysis (4.2%), hemothorax (3.0%), pneumonia (4.5%) and pericardial tamponade (0.9%), while absent of empyema. The most common complication for the lung RFA was pneumothorax. The bronchopleural fistula (BPF) was an extremely rare complication [12].

BPF was a serious complication and difficult for the treatment. Sakurai [13] and Kodama [14] performed bronchial occlusion using silicone filler and pleurodesis in three patients with a BPF. Abu-Hijleh and Blundin [15] resolved the complication through chest tube drainage. Lois M et al. [16] healed the air leaks through a chest tube to produce a pleural symphysis for management of persistent air leaks. In this report, thoracotomy was given with satisfied result. BPF also could be confirmed by bronchoscopy which should be performed as early as possible. We hypothesized that RFA damaged lung tumors and caused a tumoral necrosis hole. The hole resulted in the delayed BPF and ended in empyema subsequently (Figure 2).

In early reports, few patients received thoractomy after RFA and the data about pathological changes after RFA were rare [17]. This patient, to our surprise, the postoperative pathology confirmed a multiple small foci differentiated adenocarcinoma, partial bronchiolar - alveolar carcinoma, 0.5 cm away around the hole at the same time. It indicated that tumors existed surroundings the primary tumor 1 month after RFA. Does that suggest that the primary tumor had satellite lesions, or it was caused by the RFA treatment? Was it the reason of tumor local progression? This finding would be greatly helpful for understanding the pathological changes after RFA and even for the treatment of RFA. Should a larger ablation range be given around the tumor, or improved RFA equipment be used for the ablation. It suggested that radiofrequency electrodes might be placed in the addition area more than 1 cm away surrounded the tumor mass at least, repeated RFA and expand the range of treatment might be helpful.

## Conclusion

In conclusion, diagnose and treatments of the rare complication of BPF were difficult. Surgery was a good option for BPF especially when empyema occurred. Understanding the limitations of radiofrequency may help to decrease the risks for complications, and the larger field of ablation might be helpful for delaying the tumor local progression.

## Consent

Written informed consent was obtained from the patient for publication of this case report and any accompanying images. A copy of the written consent is available for review by the Editor-in-Chief of this journal.

## Competing interests

The authors declare that they have no competing interests.

## Authors’ contributions

WL and QL drafted the manuscript. YH arranged pictures. LH and YZ cared for the patient and provided clinical information. XL was responsible for the critical revision of the manuscript. All authors read and approved the final manuscript.
